# Supplementary material for: Gestational Iron Supplementation Improves Fetal Outcomes in a Rat Model of Prenatal Alcohol Exposure
Source: Nutrients. 2022 Apr 15;14(8):1653. doi: 10.3390/nu14081653 (PMC9025692; doi:10.3390/nu14081653)
Supplement: Supplementary file 1 [file nutrients-14-01653-s001.zip › nutrients-1619792-supplementary.pdf]

Gestational Iron Supplementation Improves Fetal Outcomes in a Rat Model of Prenatal Alcohol Exposure

Kaylee K. Helfrich [1,2], Nipun Saini [1,*], Sze Ting (Cecilia) Kwan [1], Olivia C. Rivera [1], Rachel Hodges [1] and Susan M. Smith [1,2,*]

SUPPLEMENTARY MATERIAL

Supplementary Tables (Table S1 – Table S5)

Table S1: Experimental diet composition

**Ingredient**	**Amount** (g/kg diet)
Casein, low Cu & Fe	200.0
DL-Methionine	3.0
Sucrose	549.49
Corn Starch	150.0
Corn Oil	50.0
Mineral Mix, Fe Deficient[a,*]	35.0
Vitamin Mix, AIN-76A[b,*]	10.0
Choline Bitartrate	2.0
Ethoxyquin, antioxidant	0.01
Ferrous Sulfate, heptahydrate	0.5

TD.130338 (Harlan-Teklad, Madison, WI). Provides 17.8% kcal from protein, 70.4% kcal from carbohydrate, 11.8% kcal from fat, and 4.0 kcal/g.

[a]TD.81062 (Harlan-Teklad, Madison, WI)

[b]TD.40077 (Harlan-Teklad, Madison, WI)

[*] American Institute of Nutrition. Report of the American Institute of Nutrition ad hoc committee on standards for nutritional studies. J Nutr 1977;107:1340–48.

Table S2: Maternal feces 24-hour mineral content

	CON	ALC	CON + Iron	ALC + Iron	***P* value**		
					**ALC**	**Iron**	**ALC x Iron**
Barium	0.015 ± 0.011[ab]	0.027 ± 0.009[b]	0.011 ± 0.007[a]	0.023 ± 0.016[ab]	**0.005**	0.207	0.675
Calcium	52.01 ± 14.16	47.92 ± 22.44	59.85 ± 22.93	53.86 ± 28.66	0.517	0.378	0.903
Chromium	0.033 ± 0.010	0.030 ± 0.016	0.046 ± 0.019	0.034 ± 0.021	0.101	0.341	0.538
Copper	0.055 ± 0.017	0.052 ± 0.032	0.091 ± 0.048	0.063 ± 0.040	Overall *P* = 0.248		
Potassium	0.87 ± 0.52[a]	1.95 ± 1.65[a]	1.09 ± 0.70[a]	1.65 ± 1.20[a]	**0.009**	0.996	0.508
Magnesium	2.97 ± 0.83	2.69 ± 1.33	3.58 ± 1.34	2.91 ± 1.88	0.328	0.391	0.688
Manganese	0.815 ± 0.248	0.778 ± 0.345	1.085 ± 0.443	0.840 ± 0.466	Overall *P* = 0.496		
Phosphorus	24.28 ± 6.71	24.02 ± 11.61	30.27 ± 11.65	28.77 ± 16.95	Overall *P* = 0.707		
Sulfur	9.41 ± 2.92	8.84 ± 3.23	13.64 ± 6.18	10.95 ± 6.27	0.228	0.184	0.503
Strontium	0.020 ± 0.011[a]	0.029 ± 0.006[a]	0.019 ± 0.008[a]	0.026 ± 0.014[a]	**0.039**	0.580	0.868
Zinc	0.461 ± 0.139	0.428 ± 0.194	0.647 ± 0.296	0.571 ± 0.367	Overall *P* = 0.356		

All elements are in units of total µg element in 24 hours of feces. Values are means ± SD. Elements are shown here if they were above the detection limit of ICPOES. Means that do not share a common superscript letter differ at $P<0.05$. Significant *P*-values are bolded. ALC, alcohol exposed; CON, control.

Table S3: Maternal liver mineral content

	CON	ALC	CON + Iron	ALC + Iron	***P* value**		
					**ALC**	**Iron**	**ALC x Iron**
Calcium	24.7 ± 2.3	27.4 ± 4.9	25.3 ± 5.0	26.5 ± 4.1	0.200	0.913	0.608
Copper	3.4 ± 0.2[a]	2.8 ± 0.2[b]	3.1 ± 0.3[ab]	2.7 ± 0.2[b]	**<0.001**	0.084	0.277
Potassium	3308 ± 141	3258 ± 188	3238 ± 299	3264 ± 145	0.869	0.647	0.588
Magnesium	210 ± 8	201 ± 15	203 ± 19	197 ± 8	0.116	0.205	0.710
Manganese	2.3 ± 0.1	2.3 ± 0.3	2.2 ± 0.3	2.3 ± 0.2	0.476	0.308	0.750
Sodium	748 ± 85	695 ± 77	718 ± 85	747 ± 108	0.694	0.735	0.189
Phosphorus	3157 ± 116[a]	2896 ± 183[b]	3005 ± 324[ab]	2895 ± 99[b]	**0.011**	0.272	0.273
Sulfur	5741 ± 195[a]	5226 ± 350[b]	5325 ± 516[ab]	5095 ± 182[b]	**0.003**	**0.024**	0.227
Zinc	22.6 ± 0.45	21.5 ± 2.1	20.7 ± 1.9	21.4 ± 1.5	0.715	0.072	0.116

All elements are in units of µg element/g wet weight liver. Values are means ± SD. Elements are shown here if they were above the detection limit of ICPOES. Means that do not share a common superscript letter differ at P<0.05. Significant *P*-values are bolded. ALC, alcohol exposed; CON, control.

Table S4: Fetal liver mineral content

	CON	ALC	CON + Iron	ALC + Iron	***P* value**		
					**ALC**	**Iron**	**ALC x Iron**
Calcium	44 ± 5	44 ± 5	44 ± 4	46 ± 5	0.385	0.955	0.887
Copper	8.5 ± 2.3	9.4 ± 2.8	9.6 ± 2.7	9.0 ± 3.5	0.600	0.703	0.111
Magnesium	153 ± 9[ab]	158 ± 8[a]	149 ± 9[b]	158 ± 8[ab]	**0.009**	0.356	0.975
Manganese	0.27 ± 0.05[a]	0.38 ± 0.13[b]	0.25 ± 0.04[a]	0.30 ± 0.06[a]	**<0.001**	**0.007**	0.156
Sodium	883 ± 116	926 ± 122	919 ± 93	894 ± 87	0.775	0.961	0.301
Zinc	44 ± 5	50 ± 11	45 ± 6	45 ± 5	0.192	0.352	0.137

All elements are in units of µg element/g wet weight liver. Values are means ± SD. Elements are shown here if they were above the detection limit of ICPOES. Data from males and females were combined since there was no effect of sex on mineral content. Means that do not share a common superscript letter differ at P<0.05. Significant *P*-values are bolded. ALC, alcohol exposed; CON, control.

Table S5: Fetal brain mineral content

	CON	ALC	CON + Iron	ALC + Iron	*P* value		
					**ALC**	**Iron**	**ALC x Iron**
Calcium	52 ± 4	50 ± 2	52 ± 3	51 ± 3	0.080	0.260	0.656
Copper	0.42 ± 0.04[a]	0.49 ± 0.06[b]	0.45 ± 0.05[ab]	0.48 ± 0.08[b]	**<0.001**	0.491	0.139
Magnesium	149 ±5	149 ± 6	148 ± 6	153 ± 8	0.176	0.458	0.171
Manganese	0.20 ± 0.02[a]	0.25 ± 0.04[b]	0.19 ± 0.02[a]	0.23 ± 0.03[b]	**<0.001**	**0.016**	0.325
Sodium	1172 ± 38	1176 ± 42	1205 ± 71	1197 ± 59	0.908	0.050	0.665
Zinc	9.4 ± 0.5	9.3 ± 0.5	9.2 ± 0.4	9.5 ± 0.6	0.256	0.883	0.175

All elements are in units of µg element/g wet weight brain. Values are means ± SD. Elements are shown here if they were above the detection limit of ICPOES. Data from males and females were combined since there was no effect of sex on mineral content. Means that do not share a common superscript letter differ at P<0.05. Significant *P*-values are bolded. ALC, alcohol exposed; CON, control.
